# Single-nucleotide polymorphisms in *FABP4* gene associated with growth traits in Egyptian sheep

**DOI:** 10.14202/vetworld.2020.1126-1132

**Published:** 2020-06-17

**Authors:** Heba Ibrahim Shafey, Karima Fathy Mahrous, Amal Ahmed Mohamed Hassan, Hossam Eldin Rushdi, Mohamed Abd El-Aziz Mohamed Ibrahim

**Affiliations:** 1 Department of Cell Biology, National Research Centre, Dokki, 12622 Giza, Egypt; 2 Department of Animal Production, Faculty of Agriculture, Cairo University, 12613 Giza, Egypt

**Keywords:** body weight, *FABP4* gene, growth rate, sheep, single-nucleotide polymorphisms

## Abstract

**Aim:**

The present study was performed to assess the association of single-nucleotide polymorphisms (SNPs) in the fatty acid-binding protein 4 (*FABP4*) gene with birth weight (BW), final weight (FW), and average daily gain (ADG) in three Egyptian sheep breeds.

**Materials and Methods:**

Genomic DNA was extracted from the blood samples of 50 male and female individuals representing Ossimi, Rahmani, and Barki sheep breeds. A 407 bp nucleotide (nt) segment from the first intron of *FABP4* was amplified by polymerase chain reaction, sequenced, and analyzed in the different samples.

**Results:**

Sequence analysis of the determined segment (407 bp) revealed four SNPs (all transition types) at nt position 372 (CP011894.1:g.57605471) A>G, nt position 211 (CP011894.1:g.57605632) A>G, nt position 143 (CP011894.1:g.57605700) T>C, and nt position 111 (CP011894.1:g.57605732) T>C. The allelic and genotypic frequencies for the identified SNPs in the sheep breeds were calculated. At nt positions 372 and 211, two alleles were identified (A and G). Only two genotypes were present at nt position 372 (AA and AG), while three genotypes were present at nt position 211 (AA, AG, and GG). Two alleles (T and C) and three identified genotypes (TT, TC, and CC) were detected at nt positions 143 and 111. Analysis of the results revealed that AA genotype at nt position 372 is associated with higher estimates for BW, FW, and ADG when compared to all the other genotypes. Very high correlation coefficients were found between the genotypes 143-TT and 111-TT and also between 143-TC and 111-TC. The genotypes 372-AG, 211-GG, 211-AA, 143-TT, 143-CC, 111-TT, 111-TC, and 111-CC were associated with negative effects on BW, FW, and ADG.

**Conclusion:**

The detection of four SNPs in a partial sequence of the Egyptian ovine *FABP4* gene intron 1 reflected that this gene harbors substantial diversity. In addition, a novel SNP at nt position 372 (CP011894.1:g.57605471) A>G was associated with higher estimates for BW, FW, and ADG.

## Introduction

Since domestication in the 15^th^ century, sheep is one of the most commonly recognized farm animal species worldwide. Sheep is an important agricultural animal and food source, especially in developing countries. Carcass composition is strongly related to sheep fat content. However, fat deposition in animals may be beneficial or undesirable depending on its quantity, location, and chemical composition. With the exception of intramuscular fat (IMF), excessive fat deposition has been recognized as detrimental to carcass quality and a causative agent of health risk to humans [1]. The IMF content, which is known as the total lipids extracted from muscle, has become one of the most important indicators of meat quality [2,3]. IMF is also associated with the expression of lipogenic generation [4]. Fatty acid-binding proteins (*FABPs*) are a family of homologous cytoplasmic low-molecular-mass proteins that are expressed in a highly tissue-specific manner and play an integral role in the balance between lipid and carbohydrate metabolism. Moreover, *FABP*s mediate fatty acid (FA) and/or hydrophobic ligand uptake, transport, and targeting within respective tissues. The mechanisms underlying these actions can give rise to both passive diffusional uptake and protein-mediated transmembrane transport of FAs. Nine types of *FABPs* have been identified in the mammalian *FABP* family [5,6]. Different types of *FABPs* are expressed in most mammalian tissues [7,8]. These hydrophobic ligand-binding proteins are encoded by a supergene family (*FABP* genes) [3,9].

*FABP4* is a member of the FA-binding protein family. It is also known as adipocyte FA-binding protein (A-FABP) because it has been implicated in lipid metabolism in adipose tissue through regulating gene expression of enzymes involved in lipid ­metabolism and/or by transporting FAs [3,8]. This protein has a high affinity to FAs, participates in the transport of long-chain FAs, and is more highly expressed in mature adipocytes than other cells [10]. In bovine species, the *FABP4* gene polymorphism has been associated with marbling, IMF, and meat quality [11,12]. However, *FABP4* polymorphism *Msp* A1I previously identified in three Nellore lines of Zebu cattle was not significantly associated with evaluated growth and carcass traits [13]. The FABP4 protein content was also used as a marker of IMF content accretion in the *longissimus thoracis* muscle of sheep [14]. The nucleotide polymorphisms in *FABP4* exons 1 and 2 and introns 1 and 2 affected growth and meat production traits in New Zealand Romney sheep [15]. Xu *et al*. [13] also reported a single-nucleotide polymorphism (SNP) in *FABP4* intron 1 that affects meat tenderness and IMF content in Chinese local sheep breeds. However, information regarding genes affecting growth performance of Egyptian sheep are still scarce. The three major Egyptian sheep breeds are Barki, Ossimi, and Rahmani, representing about 65% of the total sheep population. They are well adapted to local environmental conditions, fat-tailed, small to medium in size, and have a twinning rate from 1.05 to 1.30. Barki sheep are reported to have the lowest birth and mature body weights, while the Rahmani breed has the highest body weights [16].

In the present study, we used the three main Egyptian sheep breeds (Ossimi, Rahmani, and Barki) to investigate the polymorphisms in intron 1 of the ovine *FABP4* gene and to assess the potential association of SNPs detected in this gene with growth traits.

## Materials and Methods

### Ethical approval and informed consent

All necessary ethical and administrative approvals for performing this study have been obtained from the formal representatives of Faculty of Agriculture, Cairo University (CU). The collection of the blood samples was carried out by the veterinarian staff of the Agricultural Experiment Station of CU.

### Study period

The samples were collected from September 2018 to November 2018. DNA isolation, PCR and Sequence analysis were carried out from December 2018 to August 2019.

### Animals and samples collection

The current study was performed on 50 male and female individuals from Ossimi (14), Rahmani (18), and Barki (18) sheep breeds, where rams and ewes were assumed to be unrelated. The three breeds were reared as an experimental flock managed by the Agricultural Experiment Station, Faculty of Agriculture, Cairo University, Giza, Egypt. According to average daily gain (ADG), animals were classified into two distinct growth groups (low and high). Phenotypic records analyzed in the present study involved three traits: Birth weight (BW), FW at slaughter (FW), and ADG. Blood samples (5 ml) were taken from the jugular vein into vacuum tubes containing 0.25% ethylenediaminetetraacetic acid as an anticoagulant. Samples were shipped on dry ice for DNA extraction at the Laboratory of Cell Biology, National Research Centre, Giza, Egypt.

### DNA extraction

Genomic DNA was extracted from whole blood as described by Miller *et al*. [17]. DNA concentration and purity were determined using a UV spectrophotometer, Thermo Scientific, at optical densities of 260 and 280 nm.

### Polymerase chain reaction (PCR)

A DNA fragment covering 407 bp from the *FABP4* first intron was selected for PCR amplification according to the previous study of Xu *et al*. [14]. A 20 ml PCR cocktail consisted of 20 pmol forward (5’-TGGTTATTCAAGAAGCAA-3’) and reverse (5’- ATCTAAGAAAGAGCAGGC-3’) primers (HVD Life Sciences), 0.2 mM dNTPs, and 1.25 U of Taq DNA polymerase (Fermentas). The cocktail was aliquoted into PCR tubes with 100 ng of ovine DNA. The PCR cycle conditions were initial denaturation for 5 min at 94°C, followed by 35 cycles of denaturation at 94°C, annealing at 60°C, and extension at 72°C, each step for 1 min and the final extension for 5 min at 72°C. The amplification was verified by electrophoresis on 2% agarose (GIBCO, BRL, England) gel (w/v) in 1× TBE buffer. The size of PCR product was measured using a 100 bp ladder (Gene Ruler, Fermentas). The gel was stained with ethidium bromide and visualized using a UV transilluminator.

### Sequence analysis

The PCR products were purified and sequenced (Macrogen Incorporation, Seoul, South Korea). Sequence analysis and alignment to reveal nucleotide substitutions were carried out using nucleotide blast at CLUSTALW (http://www.genome.jp/tools/clustalw/) and NCBI (http://blast.ncbi.nlm.nih.gov/Blast.cgi) programs [18,19]. The SNP positions on sheep genomic DNA were identified based on the ovine chromosome 9 sequence in GenBank (accession number CP011894.1).

### Statistical analysis

Population deviation from Hardy–Weinberg equilibrium was verified by a Chi-square test. Genotypic and allelic frequencies for each polymorphic locus were calculated using Microsoft Excel data analysis add-on software XLSTAT [20]. This software was also applied to determine the significance of the fixed effects (breed, sex, and growth class) on the studied traits (BW, FW, and ADG). *A* *priori*, genotypes were considered to be independent in all analyses performed. Association analysis of SNPs in the ovine *FABP4* gene was performed separately for BW, FW, and ADG traits in the three sheep breeds studied. The model used to analyze the data was as follows:

Y_ijk_ = *μ* +G_i_ + B_j_ + S_k_ + ɛ_ijkl_

Where,

Y_ijk_ = the trait measured on each of the ijkl^th^ animal,

*μ* = the overall population mean,

G_i_ = the fixed effect associated with i^th^ genotype,

B_j_ = the fixed effect of j^th^ breed (j = 1, 2, 3), where 1 = Barki, 2 = Ossimi, and 3 = Rahmani,

S_k_ = the fixed effect of k^th^ sex (k = 1, 2), where 1 = female and 2 = male, and

ɛ_ijkl_ = random residual error.

Preliminary analysis of data showed that the interaction effects between two factors were non-significant. Therefore, interaction was excluded from the final statistical analysis. Effects associated with year of birth and season of birth were not included in the linear model, as the initial statistical analysis revealed that these effects did not significantly influence BW, FW, and ADG.

Taking into consideration the small sample size, a partial least squares regression procedure was used to create a set of h components with h<p (starting from a table with n observations described by p variables). The determination of the number of components to keep is often based on a criterion that includes cross-validation [20].

## Results and Discussion

Progress in molecular biology in the past three decades has made possible identifying genomic regions responsible for phenotypic variation in meat production traits. Several reports on SNP associations with meat traits have emerged in various livestock species [11-14,21-23]. We investigated polymorphisms in intron 1 of the Egyptian ovine *FABP4* gene and assessed the potential association of SNPs in this region of the gene with growth traits.

### Statistical analysis of growth phenotypic data

Details of the traits analyzed in the present study are shown in Table-1. Breed of the animal affected BW, FW, and ADG significantly (p<0.001).

**Table-1:** Descriptive statistics for phenotypic data.

Trait	Minimum	Maximum	Mean	Standard deviation
Birth weight (kg)	2.5	4.1	3.212	0.371
Final weight (kg)	37	63	47.228	5.947
Average daily gain (g/day)	68	142	94.28	16.905

The Ossimi breed had the highest BW followed by the Rahmani breed, but they did not differ significantly from each other (Table-2). The two breeds were significantly (p<0.01) heavier at birth compared to the Barki breed. The FW of the Rahmani breed was significantly (p<0.01) higher than that of the Ossimi and Barki breeds. However, the difference between the last two breeds was not significant. The three sheep breeds did not show significant differences from each other in ADG. Ossimi sheep exhibited the highest ADG followed by Rahmani and Barki sheep, respectively. In respect to sex of the animals, males differed significantly from females for BW (p<0.05), FW (p<0.01), and ADG (p<0.01), as shown in Table-2. Esenbuga and Dayıoğlu [24] reported that sex has affected BW of Awassi and Red Karman lambs significantly. Babar *et al*. [25]indicated that BW in Lohi sheep was significantly influenced by sex. In contrast, Sahani *et al*. [26] and Guevara *et al*. [27] reported that sex of animal had no significant effect on BW of Marwari and Pelibuey X Wiltshire Horn lambs.

**Table-2:** Least squares means of birth weight, final weight, and average daily gain for Ossimi, Rahmani, and Barki sheep breeds.

Item	Birth weight (kg)	Final weight (kg)	Average daily gain (g/day)
Breed			
Ossimi	3.412^a^	47.604^a^	99.016^a^
Rahmani	3.334^a^	50.760^b^	96.339^a^
Barki	3.031^b^	45.464^a^	95.485^a^
Sex			
Males	3.353^a^	50.076^a^	104.526^a^
Females	3.165^b^	45.809^b^	89.367^b^
Growth class			
High	3.467^a^	51.550^a^	106.417^a^
Low	3.051^b^	44.335^b^	87.477^b^

Estimates with different superscripts differ significantly from each other at p<0.05.

**Figure-1:**
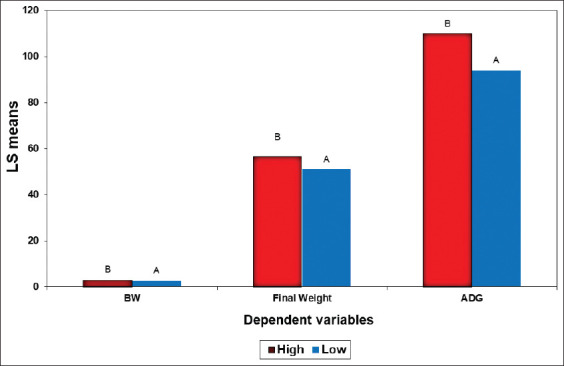
Differences between high and low classes in the studied traits genotyping by single-nucleotide polymorphisms.

Regarding growth class, differences between high and low classes were significant (p<0.001) in all studied traits. The effect of growth class was highly significant (p<0.001) in the statistical model applied. As shown in Figure-1, the small difference between the two classes at birth was converted to a larger difference at the FW. This indicated the importance of incorporating BW as a selection objective in sheep breeding programs to improve meat production traits.

### Genotyping by SNPs

A DNA fragment (407 bp) from intron 1 of the *FABP4* gene was amplified in sheep samples with high and low growth potential. The DNA fragments were purified and sequenced for SNP detection. Multiple sequence alignment for the obtained sequences along with the homologous sequences in the Genbank databases was performed to reveal differences in nucleotide sequences. Then, the obtained sequences were submitted and approved; the accession numbers registered were from MN397754 to MN397778.

**Figure-2:**
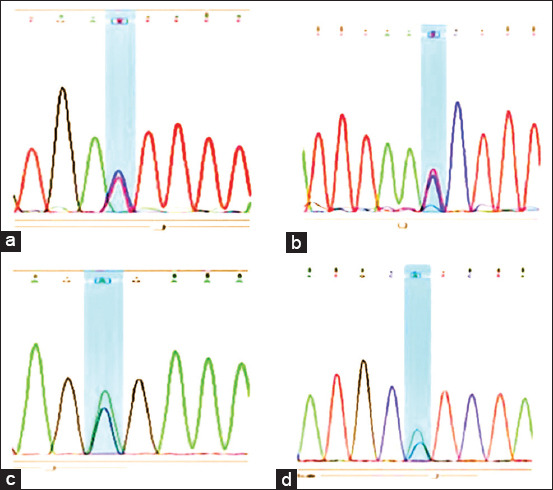
Representatives of the four detected single-nucleotide polymorphisms in heterozygous samples (a = nt. 111, b = nt. 143, c = nt. 211, d = nt. 372).

The sequence analysis of the investigated segment in the present study produced four nucleotide polymorphisms at nt position 372 (CP011894.1:g.57605471) A>G, nt position 211 (CP011894.1:g.57605632) A>G, nt position 143 (CP011894.1:g.57605700) T>C, and nt position 111 (CP011894.1:g.57605732) T>C in the analyzed samples of Ossimi, Rahmani, and Barki breeds. Representatives of the detected SNPs are shown in Figure-2. The identified polymorphisms are transitions. Allelic and genotypic frequencies at each of the four nucleotide positions are presented in Table-3.

**Table-3:** Allele and genotype frequencies at each of the four polymorphic nucleotide positions.

Position	Mean frequency	Breed				

		Allele frequency		Genotype frequency		
372		**A**	**G**	**AA**	**AG**	**GG**
	Ossimi	0.94	0.06	0.88	0.12	0.00
	Rahmani	1.00	0.00	1.00	0.00	0.00
	Barki	0.94	0.06	0.88	0.12	0.00
	Mean frequency	0.096	0.04	0.92	0.08	0.00
211		**A**	**G**	**AA**	**AG**	**GG**
	Ossimi	0.78	0.22	0.67	0.22	0.11
	Rahmani	0.50	0.50	0.33	0.33	0.33
	Barki	0.28	0.72	0.12	0.33	0.55
	Mean frequency	0.52	0.48	0.41	0.29	0.30
143		**T**	**C**	**TT**	**TC**	**CC**
	Ossimi	1.00	0.00	1.00	0.00	0.00
	Rahmani	0.78	0.22	0.72	0.14	0.14
	Barki	0.83	0.17	0.67	0.33	0.00
	Mean frequency	0.87	0.13	0.80	0.16	0.04
111		**T**	**C**	**TT**	**TC**	**CC**
	Ossimi	0.94	0.06	0.88	0.12	0.00
	Rahmani	0.78	0.22	0.72	0.14	0.14
	Barki	0.78	0.22	0.56	0.44	0.00
	Mean frequency	0.83	0.17	0.72	0.23	0.05

At nt position 372, two alleles were identified (A and G), and only two genotypes were present in the biological samples analyzed (AA and AG). The A allele was the most frequent and subsequently, the AA genotype was the most frequent in all breeds compared to the AG genotype (mean percentages: 92 vs. 8%, respectively). The GG genotype was absent in the sampled animals. A wide variation in frequency of A and G alleles was observed between the three sheep breeds at the nt position 211. The three identified genotypes at this position had the same frequency in Rahmani sheep, while high fluctuation in genotypic frequencies was noticed in Ossimi and Barki breeds. The mean percentages of AA, AG, and GG genotypes in all breeds were 37, 30, and 33%, respectively. Regarding nt position 143, allele T was observed in all breeds while allele C is absent in Ossimi sheep. Therefore, only one genotype (TT) was detected in this breed. Similarly, no CC genotype animals were observed in Barki sheep. In general, TT was the dominant genotype in all breeds, followed by TC and CC (mean percentages: 80, 16, and 4%, respectively). Two alleles were detected at position 111 (T and C) and three genotypes were identified in the sheep population analyzed. Genotype CC was present only in the Rahmani breed. In general, TT was the most common genotype in all breeds followed by TC and CC at this position (mean percentages: 72, 24, and 4%, respectively).

The identified SNP in the present study at nt ­position 143 (C/T) was reported before by Xu *et al*. [14] in Chinese sheep and was associated with meat quality traits. The association analysis revealed that the TT genotype produced higher tenderness (p<0.05), muscle marbling score (p<0.05), and IMF (p<0.05). Thus, the authors suggested that the genotype TT could be considered as a molecular marker for *longissimus thoracis* muscle tenderness and IMF content in Chinese sheep breeds.

The other three identified SNPs in the present study at nt positions 111, 211, and 372 were detected only in the Egyptian sheep. Detection of such novel nucleotide variations in Egyptian sheep in the *FABP4* gene illustrates genetic diversity at this locus.

### Correlation between the growth phenotypic data and detected genotypes

Table-4 demonstrates the correlation matrix of the detected SNPs at four positions of *FABP4* gene and the three performance traits studied. The correlation coefficient between BW and FW is close to one, followed by the correlations between FW-ADG and BW-ADG pairs, respectively. Considering ­correlations among genotypes, very high correlation coefficients were found between 143-TT and 111-TT pair as well as 143-TC and 111-TC pair.

**Table-4:** Correlation matrix between the genotypes identified on *FABP4* gene and performance traits studied.

Variables	372-AA	211-GG	211-AG	211-AA	143-TT	143-CT	143-CC	111-TT	111-CT	111-CC	BW	FW	ADG
372-AG	−1.000	0.086	0.114	−0.202	0.147	-0.129	-0.060	-0.473	0.525	−0.060	−0.253	−0.277	−0.165
372-AA		−0.086	−0.114	0.202	−0.147	0.129	0.060	0.473	−0.525	0.060	0.253	0.277	0.165
211-AA			−0.514	−0.514	−0.667	0.582	0.272	−0.646	0.554	0.272	−0.116	−0.152	0.023
211-AG				−0.471	0.343	−0.299	−0.140	0.237	−0.185	−0.140	0.166	0.078	0.190
211-GG					0.343	−0.299	−0.140	0.428	−0.385	−0.140	−0.046	0.079	−0.213
143-TT						−0.873	−0.408	0.802	−0.656	−0.408	−0.011	−0.037	−0.215
143-CT							−0.089	−0.700	0.777	−0.089	0.076	0.148	0.302
143-CC								−0.327	−0.115	1.000	−0.119	−0.201	−0.127
111-TT									−0.901	−0.327	0.143	0.134	−0.092
111-CT										−0.115	−0.096	−0.049	0.154
111-CC											−0.119	−0.201	−0.127
BW												0.909	0.812
FW													0.848

The genotypes 372-AG, 211-GG, 211-AA, 143-TT, 143-CC, 111-TC, and 111-CC were associated with negative effects on BW, FW, and ADG. On the other hand, the remaining genotypes may be favorable for selection as they had a positive influence on the three studied traits.

Fat percentage is a determinant factor for carcass composition and meat quality. Many genes are involved in fat metabolism [9,11,21]. Among those genes, the ovine *FABP4* gene is involved in gene expression regulation of the enzymes responsible for lipid metabolism and transportation of essential FAs [3,28]. Modern sheep breeding programs should address reducing fat deposition in lambs as a primary objective to attain better production efficiency, meat quality, and human health.

In the current study, particular genotypes have been associated with heavier FW at slaughter (372-GG, 211-GA, 211-AA, 143-TC, and 111-TT). If carriers of such genotypes also possess alleles associated with reduced fat deposition, this could be beneficial for mutton producers and consumers as well.

Table-5 demonstrates the standardized coefficients of variable importance in the projection (VIP) for each explanatory variable, including different categories such as breed, sex, and nucleotides detected in the *FABP4* gene. Sex led the list of VIP, where males had higher estimates than females. Considering animal genotypes for the *FABP4* gene, the AA genotype at nt position 372 was associated with higher estimates for BW, FW, and ADG compared to all the other genotypes. The 372-AG genotype was the second most influential factor in VIP. The genotype TC at position 111 of *FABP4* gene exhibited the smallest estimate of VIP.

**Table-5:** Standardized coefficients of VIP for the traits studied (birth weight, BW; final weight at slaughter, FW; and average daily gain, ADG).

Variable	VIP	Std. deviation	Lower bound (95%)	Upper bound (95%)
Sex – M	2.167	0.311	1.525	2.809
Sex – F	2.167	0.311	1.525	2.809
Breed – Barki	1.473	0.481	0.480	2.466
372-AA	0.892	0.595	−0.336	2.120
372-AG	0.892	0.595	−0.336	2.120
Breed – Ossimi	0.778	0.779	−0.830	2.386
Breed – Rahmani	0.745	0.801	−0.908	2.398
143-CT	0.652	0.810	−1.020	2.324
143-CC	0.573	0.564	−0.591	1.736
111-CC	0.573	0.564	−0.591	1.736
211-AG	0.540	0.853	−1.220	2.300
211-GG	0.328	0.724	−1.166	1.822
143-TT	0.317	0.529	−0.775	1.410
143-TT	0.256	0.758	−1.308	1.821
211-AA	0.202	0.629	−1.096	1.501
111-CT	0.007	0.605	−1.241	1.255

VIP=Variable importance in the projection

Polymorphism in the *FABP4* gene has been reported before in dairy sheep. Some variants of this gene were linked to milk fat content in Manchega sheep [29]. Xu *et al*. [14] also reported a SNP in *FABP4* intron 1 that affects meat tenderness and IMF content in Chinese native sheep breeds. The nucleotide polymorphisms in *FABP4* gene exons 1 and 2 and introns 1 and 2 affect growth and meat production in New Zealand Romney sheep [15]. In addition, Arora *et al*. [21] identified a SNP (g.4993 A>G) in the coding region of the *FABP3* gene associated with mutton quality across a panel of 11 phenotypically and geographically diverse Indian sheep breeds. Those identified SNPs are regarded as putative genetic markers for enhancing the rate of genetic improvement in sheep populations.

In the present study, we reported four SNPs in intron 1 of the Egyptian ovine *FABP4* gene. One of these SNPs at the nt position 211 was reported before to be associated with meat quality traits [14]. The authors suggested that the TT genotype at this position had a positive effect on sheep meat tenderness.

Out of the three novel SNPs that were detected here in the Egyptian sheep at nt positions 111, 143, and 372, the last one was associated with growth traits. The AA genotype at position 372 was associated with higher estimates for BW, FW, and ADG compared to all the other genotypes. The 372-AG genotype was the second most influential factor in VIP. The genotype TC at position 111 of *FABP4* gene exhibited the smallest estimate of VIP.

Future studies are necessary to establish the impact of the reported SNPs herein on economically important traits in Ossimi, Rahmani, and Barki sheep populations.

## Conclusion

The detection of four SNPs in intron 1 of the Egyptian ovine *FABP4* reflected that this gene harbors substantial diversity. However, further studies to assess the entire *FABP4* gene sequence in different sheep breeds are warranted. Moreover, the information gained in this study provides a preliminary indication of the functional diversity present in the major Egyptian sheep breeds. This will help to improve understanding the impact of breed on the genomic variation.

## Authors’ Contributions

All authors participated equally in the study plan and design. HER collected the biological samples and growth data. HIS and KFM isolated the nucleic acids, and carried out PCR and sequencing. AAMH, HER, and MAMI carried out statistical and molecular analyses. All authors collaborated in writing, revising, and improvement of the article for publication. All authors read and approved the final manuscript.
